# *Pseudomonas aeruginosa* infection exacerbates elastase induced lung damage: characterisation of a novel murine two-hit model of pulmonary infection

**DOI:** 10.1186/s12931-025-03334-2

**Published:** 2025-10-06

**Authors:** Carla A. Gustafsson, Sathayanarayani Srishanmuganathan, Anna V. Kelis, Mohamed-Amiin Muhidiin, Yanira Riffo-Vasquez, Carolyn Lam, Clive P. Page, Simon C. Pitchford, Richard T. Amison

**Affiliations:** Unit of Pulmonary Pharmacology, Institute of Pharmaceutical Sciences, School of Cancer & Pharmaceutical Science, King’s College London, London, SE1 9NH UK

**Keywords:** Pseudomonas aeruginosa, Infections, Pulmonary exacerbations, Cystic Fibrosis, Chronic Obstructive Pulmonary Disease

## Abstract

**Background:**

Pulmonary bacterial infections remain a hallmark of a range of respiratory diseases including Cystic Fibrosis (CF), Chronic Obstructive Pulmonary Disease (COPD) and non-CF bronchiectasis (NCFBE). Chronic persistent infections in the lung including *P. aeruginosa* are characterised by periods of stability punctuated by acute pulmonary exacerbations (PExs). Whilst animal infection models can reliably replicate acute and stable chronic infections, none currently replicate appropriate ‘acute-on-chronic’ pathophysiological backgrounds required to model PExs. Here, we aimed to establish a novel two-hit model of chronic *P. aeruginosa* infection where animals were first subjected to elastase challenge inducing pulmonary inflammation and injury reflective of the CF/COPD lung and subsequently infected with *P. aeruginosa*, utilised for its commonality across PExs in CF, COPD and NCFBE patients.

**Methods:**

Mice were challenged with 2 mg/kg Porcine Pancreatic Elastase (PPE) on days 1, 4 and 7 before infection with 1 × 10^6^ cfu/mouse *P. aeruginosa* strain RP73, 3 days post final PPE challenge. 2-, 5-, and 7- days post infection, both pulmonary microbiological and inflammatory readouts were quantified.

**Results:**

PPE induced significant pulmonary neutrophil recruitment alongside structural markers of lung injury characteristic of CF, COPD and NCFBE. In the two-hit cohort, bacterial clearance following *P. aeruginosa* infection was significantly impaired in animals first subjected to repeated elastase challenge. This was accompanied by exacerbated neutrophilic recruitment and activation, accelerating PPE induced lung damage characteristic of PExs.

**Conclusions:**

Taken together, this two-hit model of pulmonary infection with *P. aeruginosa* following PPE induced lung injury replicates key pathophysiological changes consistent with PExs relevant to CF, COPD and NCFBE patients.

## Background

Pulmonary bacterial infections are a hallmark of many respiratory diseases, including Cystic Fibrosis (CF) and Chronic Obstructive Pulmonary Disease (COPD) [1], and remain amongst the leading causes of morbidity and mortality worldwide with approximately 2.5 million deaths attributed to lower respiratory tract infections in 2019 [2]. Lower bacterial respiratory tract infections are typically classed as either acute or chronic infections. Acute infections are characterised as either community or hospital acquired pneumonia with common opportunistic pathogens including *Streptococcus pneumoniae (S. penumoniae)*,* Staphylococcus aureus (S. aureus)*,* Klebsiella penumoniae (K. penumoniae)* and *Pseudomonas aeruginosa (P. aeruginosa)* [3]. When identified early and treated appropriately in immunocompetent patients, these acute infections can respond rapidly to antibiotic therapy leaving limited residual damage to the lungs [4]. In contrast persistent and chronic infections often occur in patients with underlying respiratory disease, and are commonplace in patients with COPD, CF, and non-CF bronchiectasis (NCFBE) and are generally attributed to both gram-negative and gram-positive pathogens including *P. aeruginosa*, *S. aureus*,* S. pneumoniae* and *Haemophilus influenzae (H. influenzae)* [5–9]. Significantly these infections can persist for months or years despite the use of antibiotics [4]. The ability of these pathogens to colonise the airways leading to persistent infection is believed to be a result of inflammation induced lung damage (for example mucus hypersecretion and persistent neutrophilic inflammation) which compromises the ability of normal host defence mechanisms to maintain the sterility of the respiratory tract [1, 10, 11]. Significantly in these chronic infections, the immunologic response to airway infection does not develop into the expected adaptive response but instead manifests as a continuous neutrophilic response similar to those seen in acute infections [10] leading to progressive lung tissue damage and deterioration of pulmonary function [12, 13].

Persistent chronic infections experienced by patients with respiratory diseases such as CF, NCFBE, COPD and chronic bronchitis are characterised by periods of stability which are punctuated by periods of infection mediated acute worsening of symptoms known as acute pulmonary exacerbations (PExs). These are associated with increased respiratory deterioration, and ultimately increased morbidity and mortality [14–16]. PExs in patients with CF, COPD or NBCFBE can be due to either viral or bacterial infections, or in certain circumstances due to bacterial-viral coinfections [9, 14, 15, 17, 18]. The contribution of different pathogens to the development of acute bacterial PExs remains controversial. Previous research suggest they can be a result of a sudden increase in the bacterial density of the colonising pathogen, *in situ* clonal expansion of existing strains of the same pathogen, or through the acquisition of a new organism entirely [14], but further research is required to clarify this. However, as PEx rate and severity remains an important marker of disease severity, there remains a clear unmet need to more clearly understand the mechanisms underpinning the susceptibility to- and development of PEx.

Animal models have long been utilised to study both acute and chronic bacterial infections [19, 20]. In the context of lung infections with bacteria including *P. aeruginosa* and *S. aureus*, most acute respiratory infections are performed in either immunocompetent or neutropenic mice. Importantly immunocompetent models are limited by a high 1- to 3-day mortality, largely due to the high bacterial load that is required to prevent bacterial clearance and induce a tractable infection in immunocompetent mice [21, 22], whilst the use of neutropenic mice does not allow the contributions of the host immune response to be assessed [23]. Meanwhile, most chronic models of *P. aeruginosa* lung infection involve the inoculation of bacteria in immobilising agents such as agar/alginate beads to prevent bacterial clearance and mimic the bacterial growth observed in the mucus of CF/NCFBE/COPD patients [24–26]. Whilst these models replicate the clinical conditions associated with stable chronic infections such as the lung histopathology, neutrophil infiltration and enhanced cytokine production [19], none of these respiratory infection models in their current form replicate all of the appropriate pathological changes associated with the onset of acute PEx. This is likely due to the absence of prior lung injury or immune suppression that is observed in CF/COPD/NCFBE patients [1, 10], thereby not replicating the ‘acute-on-chronic’ disease that are hallmarks of acute PExs. These diseases are typically characterised by lung injury triggered by either damage associated molecular patterns (DAMPs) and/or pathogen associated molecular patterns (PAMPS) resulting in inappropriate inflammatory cell recruitment and activation, and subsequent secretion of enzymes such as myeloperoxidase (MPO), neutrophil elastase (NE), and matrix metallopeptidases (MMPs) [1]. These trigger extracellular matrix degradation, propagating airway damage, alongside goblet cell metaplasia and mucus hypersecretion [27–29]. These changes to lung pathophysiology impair bacterial clearance and enhance lung infection with opportunistic pathogens associated with PEx [1]. Significantly these underlying chronic inflammatory changes can be replicated using existing inflammatory models such as the cigarette-smoke model of COPD and the elastase mediated emphysema model [29–32]. Therefore, in this study, we sought to characterise a novel two-hit murine model of chronic *P. aeruginosa* infection in animals that had received elastase to induce lung injury and inflammation more reflective of the pathophysiological changes observed in the lungs of patients with CF or COPD.

## Materials and methods

### Maintenance and culture of bacteria for chronic infection

The *P. aeruginosa* strain RP73 which was originally obtained from a chronically infected CF patient attending the Medizinische Hochschule of Hannover, Germany, 16.9 years after the onset of infection was generously donated by Dr Alessandra Bragonzi from the San Raffaele Scientific Institute, Milan, Italy [33]. After initial collection and culture, bacteria were stored at −80 °C in cryovials. For all studies, a loopful of *P. aeruginosa* was taken from the − 80 °C stock and streaked onto a Trypticase Soy Agar (TSA) plate and incubated at 37 °C overnight. A single colony was then inoculated into 10 ml Trypticase Soy Broth (TSB) and incubated for 16 h at 37 °C under continuous shaking conditions (120 rpm). Post incubation the overnight culture was diluted by placing 2 OD of bacterial culture (approximately 1 × 10^9^ cfu) into 20 ml fresh TSB and incubated for 3–4 h to reach log phase of growth. Once at log phase, bacterial cultures were centrifuged at 2700 g for 20 min at 4 °C. The bacterial pellet was resuspended in sterile PBS and diluted to a working concentration of 2 × 10^7^ cfu/ml (OD 0.1).

### Animals

All animal experiments were performed in accordance with the Animals (Scientific) Procedures Act 1986 with 2012 amendment (PPL: P11C06933), with local ethical approval from King’s College London. Animal studies are reported in compliance with the ARRIVE guidelines. Male C57Bl/6 mice (8–10 weeks) were used throughout these experiments and were obtained from Charles River (Harlow, UK). Mice were housed in filter top cages in ventilated cabinets under standard conditions of 22 ± 2 °C with a 12:12 light: dark cycle. Mice had access to chow RM1(E) diet (Special Diets Service) and water *ad libitum* throughout and provided with wood shavings, shredded paper and cardboard tubes for environmental enrichment. Mice were randomly allocated to experimental groups so as not to confer any “cage bias”. All animals were provided with a minimum acclimatisation period of 7 days upon arrival before the commencement of the study.

### 2-hit murine model of pulmonary infection

**Fig. 1:**
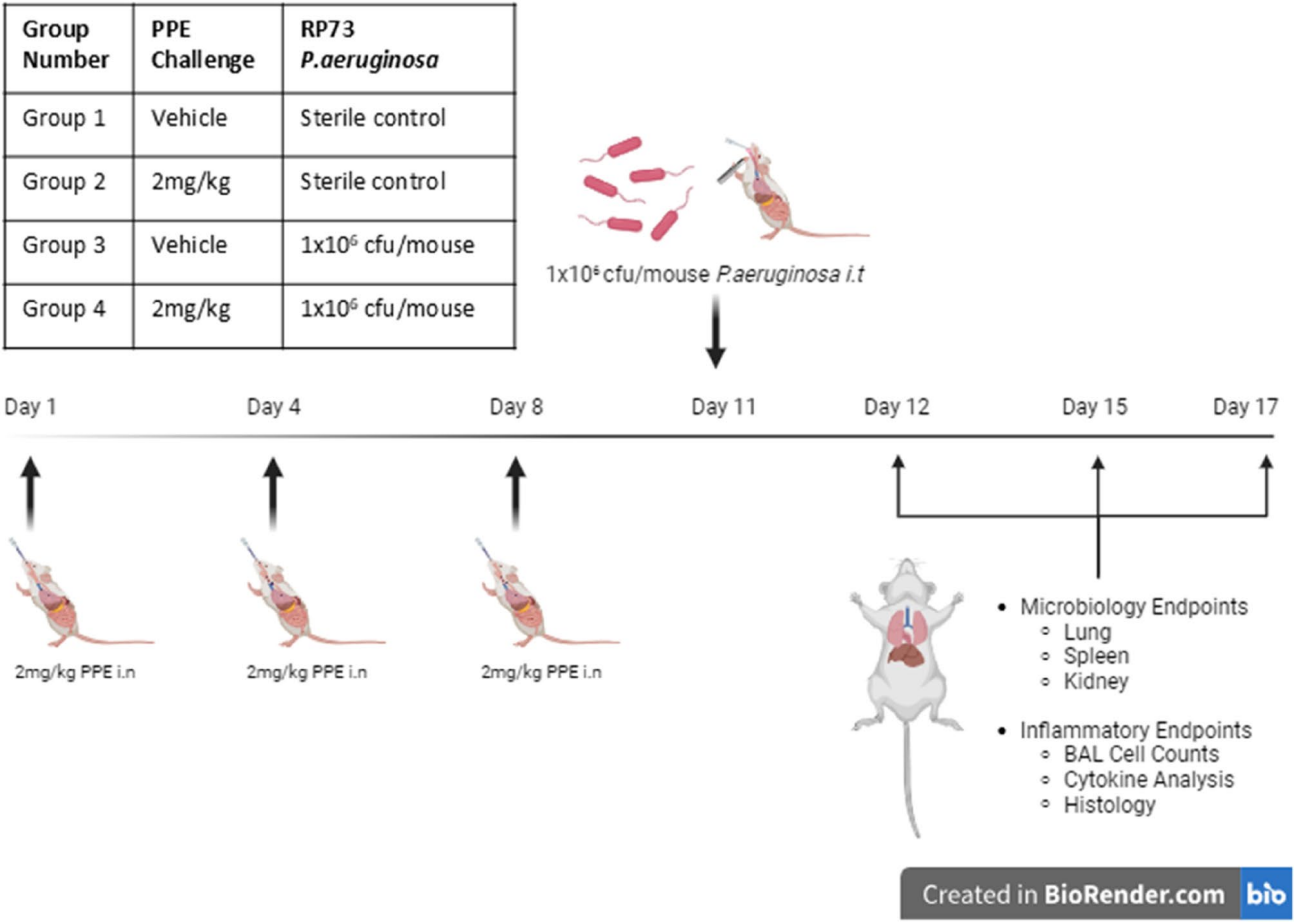
Schematic depicting the novel two-hit murine model of pulmonary infection with *P. aeruginosa* following repeated intranasal PPE challenge. C57Bl/6 mice were treated with 2 mg/kg PPE intranasally (*i.n*) on Days 1, 4 and 8; control animals were treated with vehicle only. 3-days post final aspiration, animals were inoculated with 1 × 10^6^ cfu/mouse of the *P. aeruginosa* strain RP73 or saline control via intra-tracheal (*i.t*) administration. Animals were monitored daily and groups culled at 2-, 5- and 7-days post infection (Study Days 12, 15 and 17). At each timepoint, a bronchoalveolar lavage was performed for quantification of inflammatory cell recruitment. Lungs, spleen and kidneys were aseptically removed for quantification of pulmonary bacterial load and evidence of systemic bacterial dissemination. In other studies, lungs were removed and fixed in 10% paraformaldehyde for subsequent histological analysis

On days, 1, 4 and 8 mice were challenged with 2 mg/kg Porcine Pancreatic Elastase (PPE, Sigma Aldrich) or PBS (as a control) via intranasal (*i.n*) administration under inhaled isoflurane anaesthesia. On day 10, mice were again anaesthetised, and 50 µl of 2 × 10^7^ cfu/ml (1 × 10^6^ cfu/mouse) of the *P. aeruginosa* isolate RP73 was instilled directly into the respiratory tract via intratracheal (*i.t*) dosing. After administration, the bacterial inoculum was serially diluted 1:10 in PBS and plated for bacterial enumeration to confirm the concentration administered to the mice. Sham control mice were inoculated with 50 µl sterile PBS. A Diagrammatic representation of the two-hit model alongside group allocations can be found in Fig. 1, with groups for the study being Group 1: Saline: Saline Control (Naïve), Group 2: 2 mg/kg PPE alone, Group 3: Saline: RP73, Group 4: PPE: RP73. Animals with no PPE treatment prior to infection are referred to as the ‘One-Hit Cohort’ (Group 3), whilst animals pre-treated with PPE prior to infection are referred to as the ‘Two-Hit Cohort’ (Group 4). Animals were monitored at regular intervals for signs of pain and distress, and body weight was measured daily. 2-, 5- and 7-days post infection animals were terminally euthanised using 25% w/v urethane via intra-peritoneal injection. At each time point a bronchoalveolar lavage (BAL) was performed via insertion of a 22-gauge catheter into the trachea and flushed 3 times with 0.5 ml sterile PBS. Total cell counts were performed by adding Tuerks stain in a 1:1 ratio and quantified using an Improved Neubauer chamber (Hawksley and Sons Ltd), and a bright field microscope under a 20x objective (Leica DM2000 LED). Cytospin slides were prepared and 100 µl BAL fluid added into a cytospin funnel and centrifuged at 960 rpm for 5 min under medium acceleration (Shandon, UK). Slides were then stained using Diff-Quick (Dade, Biomap, Italy). Following enumeration of inflammatory cells, BAL fluid was centrifuged and frozen at −80 °C for subsequent cytokine analysis.

### Lung, spleen and kidney harvest for microbiological and histological analysis

2-, 5- and 7- days post infection, lungs, spleen and kidneys were removed and homogenised in 2 ml sterile PBS. Tissues samples and BAL samples were serially diluted 1:10 in PBS and plated on TSA plates for bacterial enumeration. For total lung bacterial load, cfu counts were combined from both lung homogenate and BAL samples. Whilst the TSA plates are not selective for *P. aeruginosa*, colonies were identified by their large opaque and tanned colour.

In separate mice, lungs not subjected to BAL were inflated *in situ* via an intratracheal cannula in an open chest cavity with 10% (w/v) buffered formalin and immersion fixed for 48 h. Post fixation, lungs were sliced horizontally and processed into paraffin embedded blocks. De-waxed slices (5 μm thick) were prepared on a microtome.

### Histological staining

For routine histological staining, lung sections were cleared in xylene, rehydrated in decreasing concentrations of ethanol, and stained with haematoxylin and eosin (Sigma Aldrich, UK). Sections were dehydrated in increasing concentrations of ethanol, cleared in xylene, and cover-slipped with DPX. Images were captured using a brightfield microscope (DM2000 LED Bright Field Microscope, Leica Microsystems, UK) attached to a camera (DFC295 Bright Field Microscope Camera, Leica Microsystems, UK).

### Periodic acid-schiff (PAS)/Alcian blue tissue staining

For quantification of goblet cell metaplasia, a commercial PAS staining system was utilised. Briefly, sections were exposed to periodic acid for 5 min followed by submersion in sulphite water for 2 min and then washed with distilled water. Slides were subsequently submerged in Schiff’s reagents for 15 min and again washed in distilled water. Slides were then finally submerged in haematoxylin for 3 s, washed and then dehydrated as previously described. Slides were then immersed in xylene and mounted with DPX mountant and cover slipped. To determine PAS scores, images were captured under 20x magnification. Using ImageJ, the internal diameter of the airways was measured with a threshold of > 200 μm required for inclusion. A semi-quantitative mucus grading score (0–3) was used to quantify positive PAS/Alcian-blue staining for quantification of airway goblet cell metaplasia as previously described [34]. This system is based on the ratio of positively stained goblet cell area to the whole cross-sectional epithelial area. A score of 0 indicates no positive staining for goblet cells, a score of 1 indicates that ≤ 1/3 of the epithelial area was positively stained, a score 2 indicates that > 1/3 of the epithelial area was positively stained, and a score of 3 meant that ≥ 2/3 of the epithelial was positively stained. The PAS score was then obtained by averaging the scores for each group. A minimum of 12 large airways were considered per group.

### Quantification of lung injury

Two common attributes of pulmonary damage attributed to chronic bacterial lung infections are the breakdown in the alveolar structure and thickening of the alveolar septa. We therefore used two specific techniques to quantify this. Mean linear intercept (MLI) scores are a common metric for quantification of lung injury in histopathology images as it describes the mean free distance in the airspaces. We used a semi-automated measurement of the MLI adapted from methodology reported by Crowley and colleagues [35]. Briefly, 10 lung images at 20X magnification were taken per lung section using a brightfield microscope (DM2000 LED Bright Field Microscope, Leica Microsystems, UK) attached to a camera (DFC295 Bright Field Microscope Camera, Leica Microsystems, UK). Using Image J (Plug in: Measure MLI plugin for ImageJ v1.52 [35]), the initial images were converted to 8-bit images, and subsequently binarized to distinguish between airspace and lung tissue. Semi-transparent horizontal test lines were the added to the image at 100-pixel distance from each other, and a colour threshold applied to isolate discrete chords based on pixel colour. This produced an output listing all of the co-ordinates where the lung tissue intercepts each horizontal chord per image. The number of co-ordinates per chord is counted, and the ML calculated by dividing the number of co-ordinates on a chord by the length of that chord (in our studies this was 330.32 μm). The mean MLI per picture is then calculated from the total chords in in each image allowing the calculation of the mean MLI per tissue section and subsequently mean MLI per animal. Finally, we also measured the mean septal width (MSW) per animal, by measuring average septa widths on each line within each image.

### Pulmonary immunohistochemistry

For immunohistochemistry (IHC), lung sections were cleared in xylene and rehydrated in decreasing concentrations of ethanol before undergoing blocking in 4% hydrogen peroxide (Sigma Aldrich, UK). Sections underwent antigen retrieval in sodium citrate buffer (pH 6.0) for 5 min at 100 °C. Slides were blocked in 1% BSA before the addition of a rabbit anti-myeloperoxidase (MPO) antibody (BS-4943R, ThermoFisher) as a measure of pulmonary neutrophil activity. This was followed by incubation with a secondary biotinylated anti-rabbit IgG antibody (BA-1000-1.5, Vector Laboratories) and detected using an ABC detection kit. Sections underwent DAB development and a haematoxylin counterstain. Sections were dehydrated in increasing concentrations of ethanol, cleared in xylene, and cover slipped with DPX.

### Histological quantification

To quantify MPO activity, 10 nonoverlapping brightfield images were captured per lung section using a 20X objective on a Leica DM upright microscope. Using ImageJ and the “Colour_Deconvolution_2” plugin, the images were deconvoluted according to “H DAB” vectors into a haematoxylin only image (blue), DAB only image (brown), and a background image (all remaining colours). Subsequent colour thresholding was performed based on the control images to identify the required threshold for positive DAB staining. The total area of positive staining (%) per image was then quantified. The mean total area of positive staining was then quantified and expressed as a standardised value reported as the mean control-corrected area of staining per animal (%).

### Quantification of soluble inflammatory markers via ELISA

Total levels of soluble inflammatory cytokines (TNFα, IL-6, MCP-1, KC, and Neutrophil Elastase) were measured in BAL fluid and were measured by ELISA according to the manufacturer (R&D Systems).

### Statistical analysis

Data from all studies are expressed as mean (± SEM). Data were tested for normality using the Shapiro-Wilks normality test in GraphPad Prism (version 10.4.2). Data that passed normality (Elastase validations studies, Microbiology, MLI, MPO and NE) testing were analysed using a One-way Annova with Sidaks multiple comparison test. Data that was not normally distributed (Inflammatory Cells and ELISAs) were analysed using the non-parametric Kruskal-Wallis test with Dunn’s multiple comparison post-test. Data was plotted using GraphPad Prism. A P value of < 0.05 was considered significant. Statistical comparisons demonstrated using ‘*’ represent significant differences compared to Sham control (Column 1). Statistical comparisons demonstrated using ‘#’ represent significant differences between 1- and 2-hit cohorts (Columns 2 vs. 4 and 3 vs. 4).

## Results

### Pulmonary elastase challenge induces key features of lung damage

**Fig. 2:**
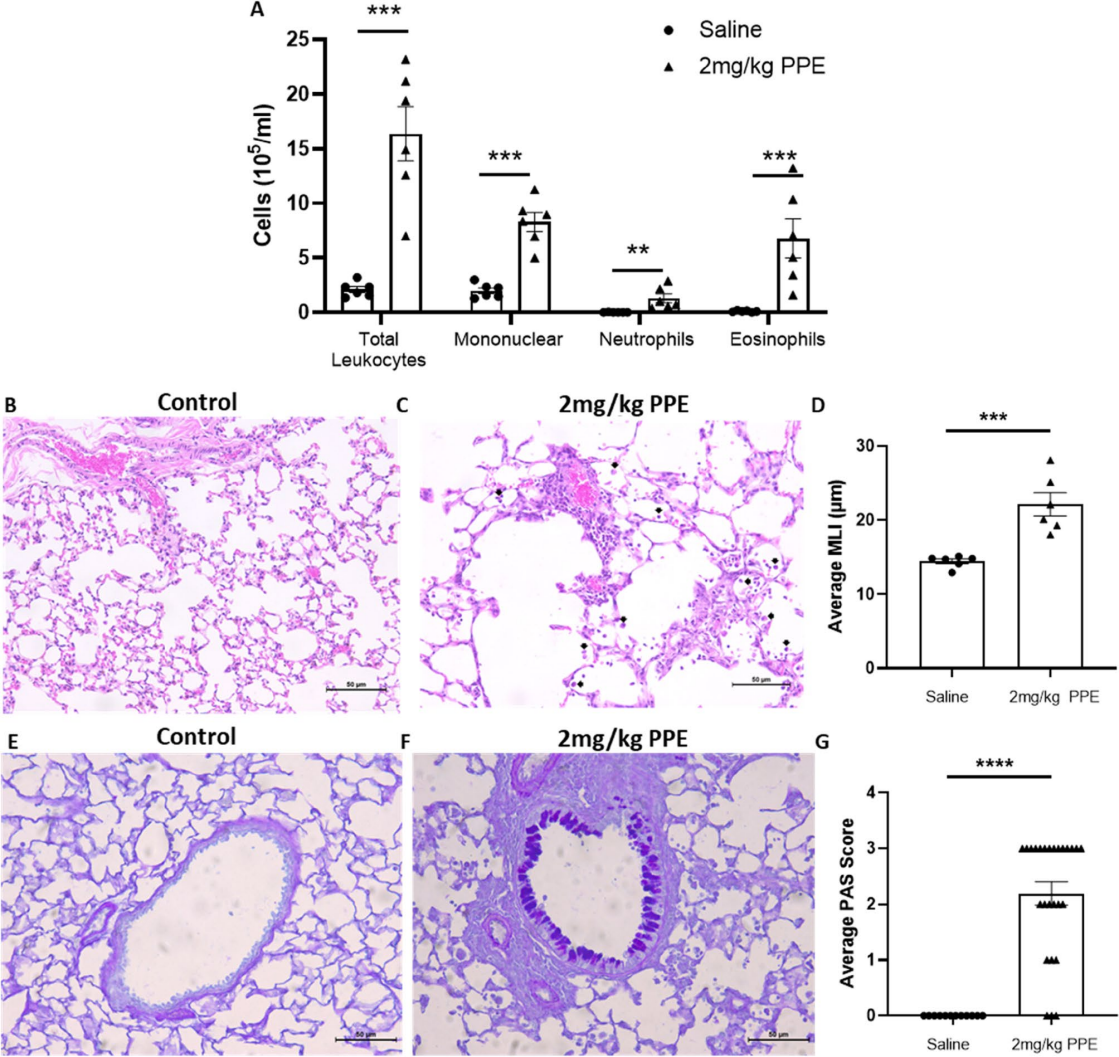
Repeated PPE intranasal challenge is associated with increased pulmonary inflammatory cell recruitment, tissue damage and goblet cell metaplasia. C57Bl/6 mice were treated with 2 mg/kg PPE *i.n* on Days 1, 4 and 8; Control animals were treated with vehicle only. 3-days post final aspiration (Day 11), a bronchoalveolar lavage was performed and both total and differential cell counts enumerated (**A**). Lungs were subsequently removed and fixed in 10% paraformaldehyde. Tissues sections were stained with either haematoxylin and eosin (H&E) (**B**: representative micrograph for vehicle control; (**C**: representative micrograph for PPE Exposed) for quantification of lung damage using the mean linear intercept (**D**) or stained with a periodic acid-Schiff (PAS)/Alcian blue stain for quantification of goblet cell metaplasia (**E**: Representative micrograph for vehicle control; (**F**: representative micrograph for PPE Exposed) and measured semi-quantitatively using a PAS scoring system (**G**). Data are represented as mean ± SEM with *n* = 6. Black arrow heads identify inflammatory cell infiltrate. Data were analysed using a Mann Whitney t-test. *** = *P* < 0.001, **** *P* < 0.0001 compared to vehicle control

Many patients with airway diseases associated with either acute/chronic bacterial infections such as COPD, CF, and NCFBE experience significant lung injury triggered by either DAMPs and/or PAMPs. This leads to inappropriate inflammatory cell recruitment, and the activation/secretion of enzymes such as MPO, NE and MMPs which trigger further extracellular matrix degradation with subsequent propagation of airway damage and goblet cell metaplasia. We therefore first examined the clinical relevance of lung damage induced through repeated elastase aspiration. As shown in Fig. 2A, repeated elastase aspiration on days 1, 4 and 8 induced significant pulmonary inflammatory cell recruitment 3-days post final aspiration (Day 11) with significant increases in neutrophils (*P* < 0.05), eosinophils (*P* < 0.05) and monocytes (*P* < 0.05) observed at Day 11. Histopathological examination of the lungs also revealed characteristic damage to the epithelial layer, leukocytic accumulation surrounding the airways and within the alveolar spaces, alongside evidence of alveolar loss and septal thickening (Fig. 2B and C). MLI methodology was used to quantify lung damage, with elastase challenge significantly increasing MLI compared to vehicle controls (Vehicle: 14.47 ± 0.33 μm vs. PPE: 22.14 ± 1.57 μm, *P* < 0.001, Fig. 2D). Finally, lungs exposed to elastase challenge demonstrated significant evidence of goblet cell metaplasia (Fig. 2E and F), as quantified by a significant increase in PAS scoring compared to vehicle control 3 days post final aspiration (Vehicle: 0.00 ± 0.00 vs. PPE: 2.19 ± 0.2,1 *P* < 0.001, Fig. 2G). When calculating the PAS score for each group a minimum airway diameter of 200 μm was required for analysis. For the control group a total of 12 airways across group met this threshold, whilst 26 airways met this threshold in the repeated PPE challenge group.

### Pulmonary challenge with elastase prior to infection with P. aeruginosa induces a chronic lung infection that persists for 7 days

Following confirmation that PPE challenge induced clinically relevant lung damage 3 days post final aspiration (Day 11), we evaluated whether animals previously challenged with PPE were more susceptible to persistent bacterial infection (following inoculation with a free bacterial suspension) when compared with healthy animals. A number of different *P.aeruginosa* strains have been ustilised to develop models of pulmonary infection, all of which replicate characteristic features of respiratory diseases such as CF and COPD. A commonly used strain in such models is the laboratory strain PAO1 which was initially isolated from a wound infection. However, PA01 is more virulent than strains isolated from patients with CF and COPD and has much faster replication [36]. Here we have used a clinical isolate of *P. aeruginosa* taken from a chronically infected 40-year-old CF patient which is able to produce biofilms both *in vitro* and *in vivo*, and has been used to model CF lung infections whilst not causing significant mortality in the mice [24, 37].

**Fig. 3:**
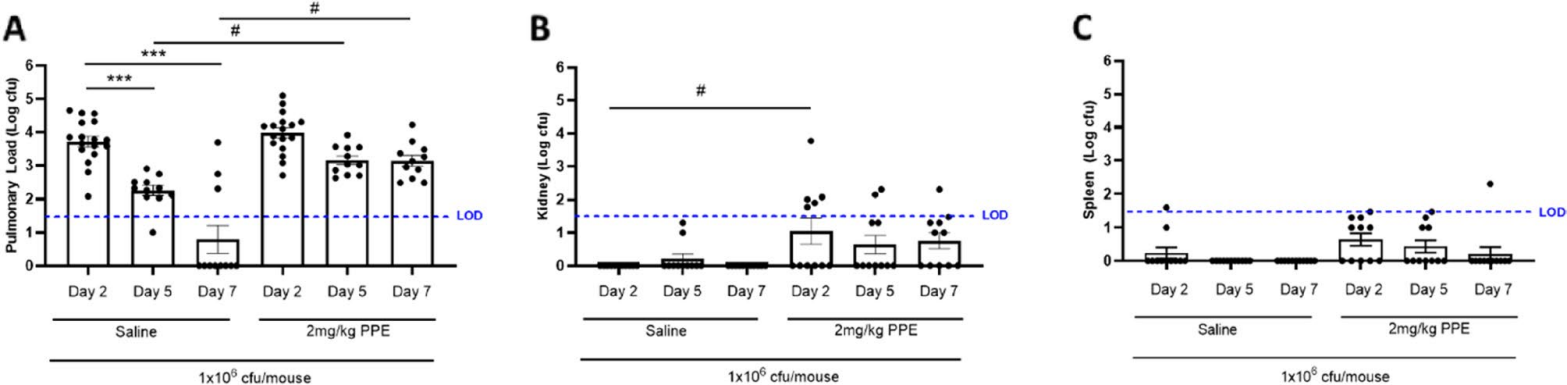
Post PPE challenge pulmonary *P. aeruginosa* infection persists for 7 days after inoculation, but not in healthy lungs. C57Bl/6 mice were treated with 2 mg/kg PPE *i.n* on Days 1, 4 and 8; Control animals were treated with vehicle only. 3-days post final aspiration animals were inoculated with 1 × 10^6^ cfu/mouse of the *P. aeruginosa* strain RP73 or saline control via *i.t* administration. Animals were culled at 2-, 5- and 7-days post infection (Study Days 12, 15 and 17). Lungs (**A)**, spleen (**B**) and kidneys (**C**) were aseptically removed for quantification of pulmonary bacterial load and evidence of systemic bacterial dissemination. Data are represented as mean Log cfu/lung ± SEM with *n* = 11–17 across 3 independent studies. Data were analysed using a one-way ANOVA and Sidaks multiple comparisons post-hoc test. * = *P* < 0.05, ** = *P* < 0.01, **** *P* < 0.0001 compared to Day 2 post infection. # = *P* < 0.05, compared between 1-hit and 2-hit cohorts

Animals were infected with 1 × 10^6^ cfu/mouse RP73 3 days post final elastase aspiration, and total pulmonary bacterial load quantified through the sum of recovered bacteria from lung homogenates and BALF in animals at 2-, 5-, and 7-days post bacterial inoculation. At 2-, 5-, and 7-days post infection, mice inoculated with sterile PBS demonstrated no evidence of infection in the lungs, kidney, or spleen (data not shown). In contrast healthy animals with no prior elastase challenge (one-hit cohort) demonstrated a peak in total pulmonary bacterial load 2 days post infection vs. Sham control (3.72 ± 0.16 log cfu/lung, *P* < 0.001) which demonstrated significant resolution over the 7 day time course, shown by a significant reduction in bacterial numbers at 5- (Day 5: 2.25 ± 0.15 log cfu/lung, *P* < 0.01) and 7- (Day 7: 0.79 ± 0.42 log cfu/lung), *P* < 0.001) days post infection compared to day 2 (Fig. 3A). Importantly bacterial numbers were below the threshold for accurate quantification 7 days post infection, suggestive of complete resolution. Animals infected with *P. aeruginosa* following prior elastase challenge (two-hit cohort) also demonstrated infection which peaked at 2-days post infection (3.98 ± 0.16 log cfu/lung, *P* < 0.0001). Importantly this was not significantly different to pulmonary bacterial numbers in infected animals with no prior lung injury. However, in contrast to animals in the 1-hit cohort, animals infected with *P. aeruginosa* in the 2-hit cohort demonstrated a persistent and sustained bacterial infection with no significant reductions in pulmonary bacterial load over the 7-day time course (Day 5: 3.16 ± 0.13 log cfu/lung, Day 7: 3.14 ± 0.16 log cfu/lung) (Fig. 3A**)**.

No significant evidence of systemic infection, as determined by bacterial recovery from the spleen or kidney, was observed in infected animals belonging to the one-hit cohort. However bacterial numbers were elevated in the kidneys of infected animals in the two-hit cohort at days 2 (*P* < 0.01) and 5 (*P* < 0.05) post infection (Fig. 3B & C).

### P. aeruginosa infection exacerbates elastase induced pulmonary leukocyte recruitment over 7 days

**Fig. 4:**
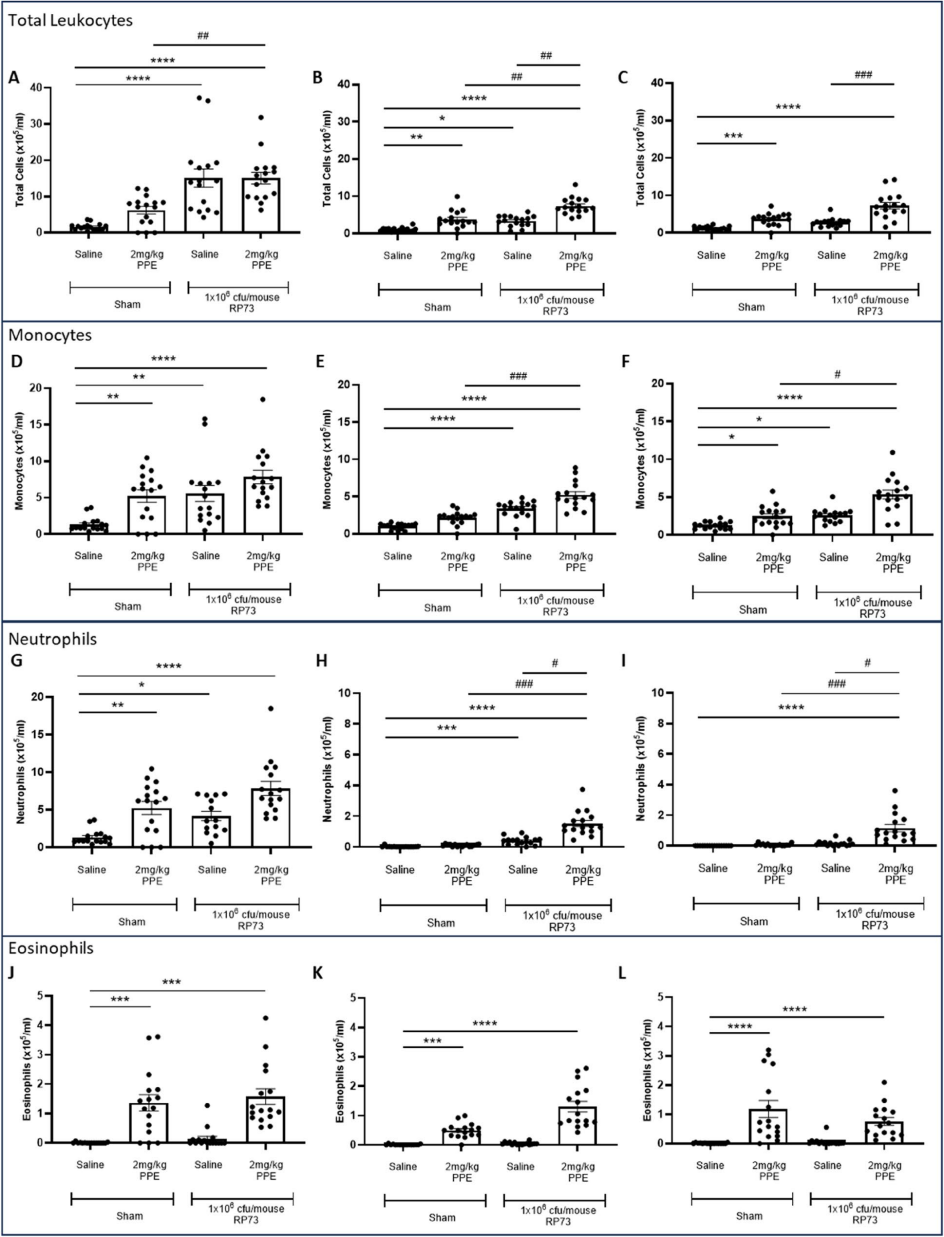
Pulmonary *P. aeruginosa* infection induces pulmonary inflammation that persists throughout 7 days of infection when inoculated following initial PPE challenge, but not in healthy lungs. C57Bl/6 mice were treated with 2 mg/kg PPE *i.n* on Days 1, 4 and 8; control animals were treated with vehicle only. 3-days post final aspiration animals were inoculated with 1 × 10^6^ cfu/mouse of the *P. aeruginosa* strain RP73 or saline control via *i.t* administration. 2- (**A**, **D**, **G** and **J**), 5- (**B**, **E**, **H** and **K**) and 7-days (**C**, **F**, **I** and **L**) post infection (Study Days 12, 15 and 17), a bronchoalveolar lavage was performed and analysed for total leukocyte counts (**A-C**), monocytes (**D-F**), neutrophils (**G-I**) and eosinophils (**J-K**). Data are represented as mean cells/ml ± SEM with *n* = 16 across 2 independent studies. Data were analysed using a Kruskal-Wallis test followed by Dunn’s multiple comparisons post-hoc test. * = *P* < 0.05, ** = *P* < 0.01, *** *P* < 0.001, **** = *P* < 0.0001 compared to vehicle control (column A). ## = *P* < 0.01, ### = *P* < 0.001 compared between 1-hit and 2-hit cohorts (column B vs. D and C vs. D)

As pulmonary bacterial infections with pathogens such as *P. aeruginosa* are regularly associated with an influx of inflammatory cells, in particular neutrophils, we next assessed infection-induced pulmonary leukocyte recruitment in both the one- and two-hit cohorts (Fig. 4). In agreement with earlier studies, repeated elastase challenge induced significant pulmonary leukocyte recruitment at 2 days post infection (*P* < 0.001, Fig. 4A) which was characterised by corresponding increases in monocytes (Fig. 4D), neutrophils (Fig. 4G) and eosinophils (Fig. 4J). Total leukocyte (Fig. 4A-C), monocyte (Fig. 4D-F) and neutrophil numbers (Fig. 4G-I) in the lung progressively reduced over the duration of the study, with numbers returning to baseline by 7 days post infection. Conversely, eosinophil numbers (Fig. 4J-L) remained significantly elevated for the duration of the study likely due to the elastase challenge.

In the one-hit cohort, both total leukocyte counts (Fig. 4A) and monocytes (Fig. 4D) in the BAL fluid were significantly elevated over naïve controls (group 1) 2-days post infection (Total: *P* < 0.0001, Monocytes: *P* < 0.01). Total leukocyte numbers subsequently reduced over the remaining time-course of infection, and whilst they remained significantly elevated at 5-days post infection (Total: *P* < 0.01, Monocytes: *P* < 0.0001, Fig. 4B & E), they had returned to basal levels by 7-days post infection (Fig. 4C & F). No changes in eosinophil levels were observed at any timepoint (Fig. 4J-L). In contrast, in the two-hit cohort, significant pulmonary leukocyte recruitment was observed at 2-days post infection (*P* < 0.001, Fig. 4A), which was characterised by corresponding increases in monocytes (*P* < 0.0001, Figure D), neutrophils (*P* < 0.0001, Fig. 4G) and eosinophils (*P* < 0.0001, Fig. 4J) versus naïve controls. Eosinophil numbers remained significantly elevated for the duration of the study, but this was not significantly different to eosinophil levels induced by PPE challenge alone. In contrast to the 1-hit cohort, whilst partial resolution of both monocyte and neutrophil numbers was observed over the 7-day time-course of infection, both monocyte (5dpi: *P* < 0.0001; 7dpi: *P* < 0.0001, Fig. 4E & F) and neutrophil (5dpi: *P* < 0.0001; 7dpi: *P* < 0.0001, Fig. 4H & I) numbers remained significantly elevated throughout the duration of the study versus naïve controls (Group 1). Both neutrophil and monocyte numbers were significantly elevated over both the PPE challenged animals (Group 2) and the one-hit cohort (Group 3) at all timepoints suggestive of exacerbated pulmonary leukocyte recruitment in the two-hit cohort (Figs. 4A-F).

### Pulmonary challenge with elastase prior to infection with P. aeruginosa suppressed pro-inflammatory cytokine release 2 days post infection

**Fig. 5:**
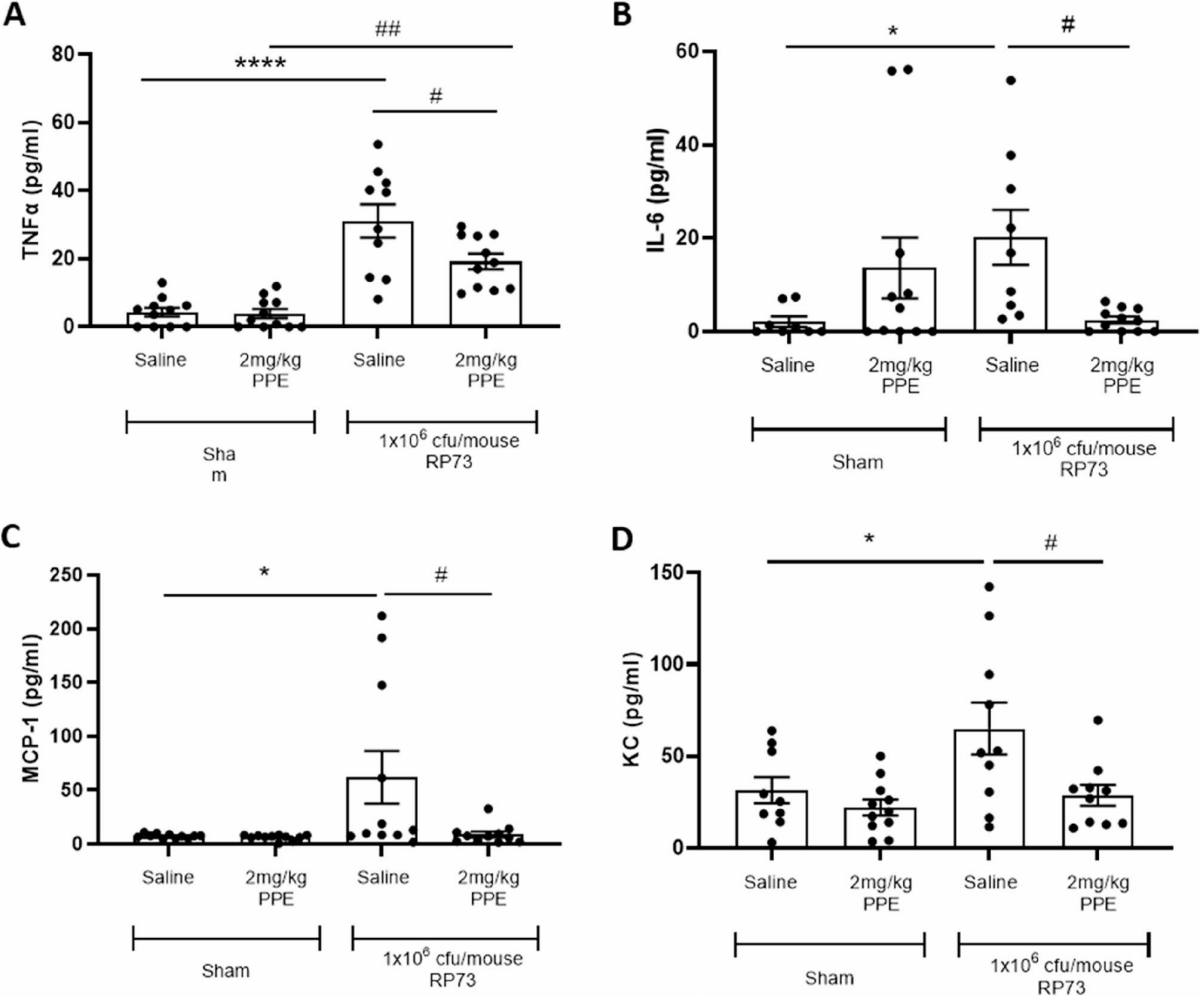
Pulmonary *P. aeruginosa* infection following intranasal PPE challenge demonstrates an impaired of pro-inflammatory cytokine release 2 days post infection. C57Bl/6 mice were treated with 2 mg/kg PPE *i.n* on Days 1, 4 and 8; control animals were treated with vehicle only. 3-days post final aspiration animals were inoculated with 1 × 10^6^ cfu/mouse of the *P. aeruginosa* strain RP73 or saline control via *i.t* administration. 2-days post infection a bronchoalveolar lavage was performed and analysed for TNFα (**A**), IL-6 (**B**), MCP-1 (**C**) and KC (**D**). Data are represented as mean pg/ml ± SEM with *n* = 11 across 2 independent studies. Data were analysed using a one-way ANOVA and Tukey’s multiple comparisons post-hoc test. * = *P* < 0.05, ** = *P* < 0.01, compared to vehicle control (column A). # = *P* < 0.05 compared between 1-hi and 2-hit cohorts (column B vs. D and C vs. D)

In these studies, we have demonstrated that infection of free bacterial inoculum in the absence of prior PPE-induced lung injury results in a resolving infection over the time-course of this study. However, when animals were infected following prior PPE challenge, both the pulmonary bacterial load and associated inflammatory cell infiltrate persisted for the duration of the study. We therefore sought to investigate whether prior PPE challenge altered the release of pro-inflammatory cytokines known to be involved in the resolution of infection. We therefore evaluated the release of TNFα (Fig. 5A**)**, IL-6 (Fig. 5B**)**, MCP-1 (Fig. 5C) and KC (murine surrogate of IL-8) (Fig. 5D**)** in BAL fluid at 2-, 5- and 7-days post infection. Cytokine titres 2-days post infection are shown in Fig. 5. Repeated challenge with PPE was not associated with any increases in either TNFα, IL-6, MCP-1, or KC 2-days post infection). In the one-hit cohort, in agreement with previous data, acute *P. aeruginosa* infection was associated with significant increases in TNFα (*P* < 0.001, Fig. 5A), MCP-1 (*P* < 0.05, Fig. 5C), and KC (*P* < 0.05, Fig. 5D). Whilst IL-6 levels were elevated, this did not reach statistical significance (Fig. 5B). In the two-hit cohort, cytokine release appeared impaired as shown by significantly reduced TNFα (*P* < 0.05, Fig. 5A), IL-6 (*P* < 0.05, Fig. 5B), MCP-1 (*P* < 0.05, Fig. 5C) and KC (*P* < 0.05, Fig. 5D) compared to the one-hit cohort. No significant changes in any measured cytokines were detected either 5- or 7-days post infection (data not shown). The observed differences in cytokine expression levels differ between groups may be due to the differences in the timing of inflammation onset, and therefore differences in the kinetics of cytokine expression.

### Pulmonary infection with P. aeruginosa exacerbates histological evidence of elastase mediated lung damage

**Fig. 6:**
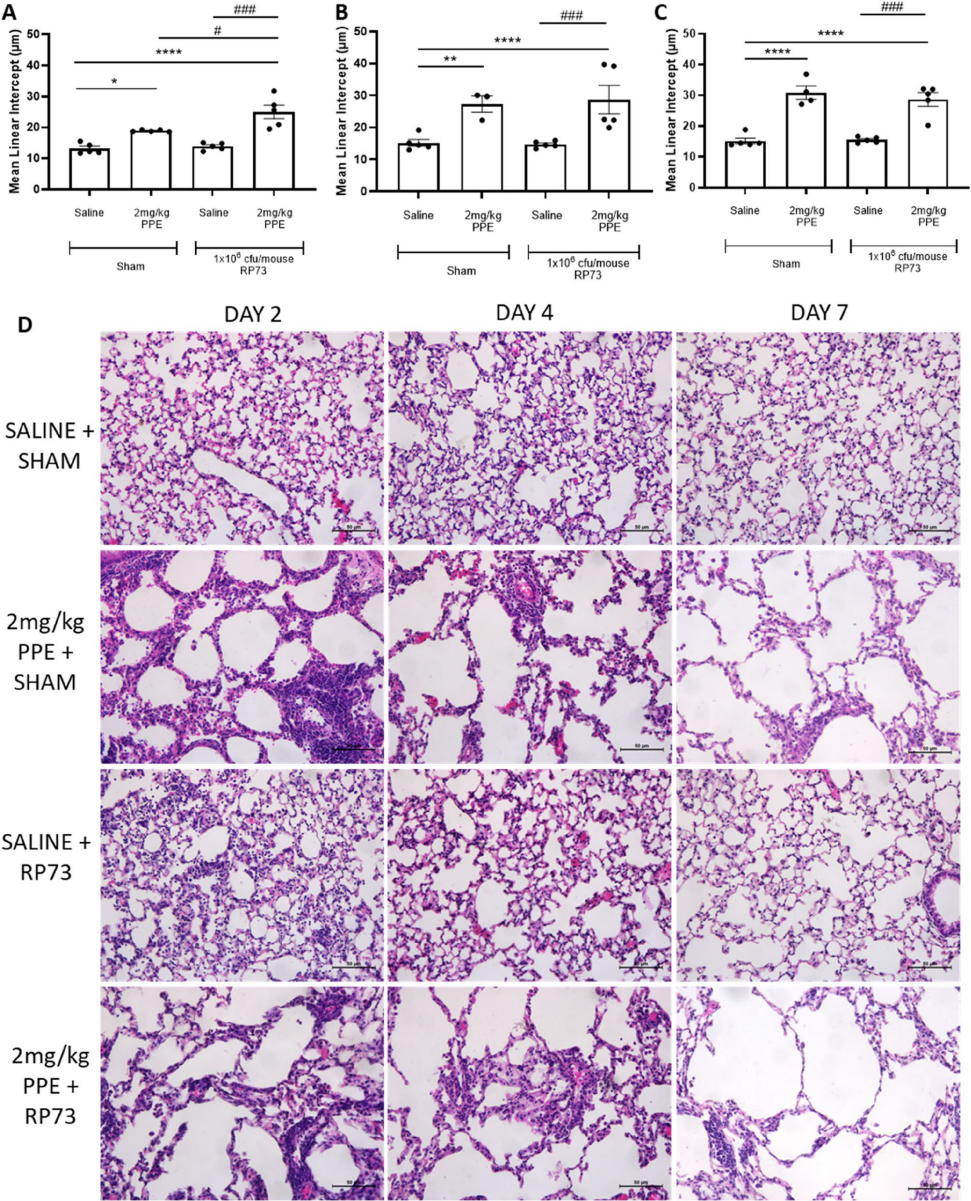
Prior Elastase Challenge exacerbates *P. aeruginosa* induced lung damage C57Bl/6 mice were treated with 2 mg/kg PPE *i.n* on Days 1, 4 and 8; control animals were treated with vehicle only. 3-days post final aspiration animals were inoculated with 1 × 10^6^ cfu/mouse of the *P. aeruginosa* strain RP73 or saline control via *i.t* administration. Lungs were removed and fixed in 10% paraformaldehyde. Tissue sections were stained with haematoxylin and eosin (H&E) and lung damage quantified using the mean linear intercept at 2- (**A**), 5- (**B**), and 7- (**C**) days post infection (Study Days 12, 15 and 17). **D**: Representative H&E micrographs from Saline: Sham, PPE: Sham, Saline:*P.aeruginosa* and PPE:*P.aeruginosa* mice at 2-, 5- and 7- days post infection. Data are represented as mean ± SEM with *n* = 3–5 from 1 independent study. Data were analysed using a one-way ANOVA and Sidaks multiple comparisons post-hoc test. * = *P* < 0.05, ** = *P* < 0.01, *** *P* < 0.001, **** = *P* < 0.0001 compared to vehicle control (column A). ## = *P* < 0.01, ### = *P* < 0.001 compared between 1-hi and 2-hit cohorts (column B vs. D and C vs. D)

Whilst chronic respiratory infections with pathogens such as *P. aeruginosa* are significant contributors to lung decline in patients with CF and COPD, the occurrence of acute infective exacerbations is one of the most important clinical events in these patients. These acute infective exacerbations are associated with accelerated lung damage, linked to further lung function decline. We therefore sought to investigate whether *P. aeruginosa* infection following the induction of clinically relevant lung damage with PPE was able to exacerbate lung damage in this model. MLI methodology was used as a measure of morphometric changes in the lungs to quantify progressive lung damage in these animals. In agreement with validation data showed in Fig. 2, repeated PPE challenge significantly increased the MLI compared to vehicle controls at all time points (2-days post infection: *P* < 0.05, 5-days post infection: *P* < 0.01, 7-days post infection: *P* < 0.001, Fig. 6A-C). The extent of lung damage increased throughout the duration of the study with a significantly higher MLI measured at 7-days post infection compared to 2-days post infection (*P* < 0.01, Fig. 6A-C). Pulmonary infection with *P. aeruginosa* alone (one-hit cohort) showed no significant increases in MLI at any timepoint. However, in the two-hit cohort, PPE-induced increases in MLI were accelerated compared to PPE challenge alone, as shown by a significant increase in MLI detected at 2-days post infection when compared to the one-hit cohorts (*P* < 0.05, Fig. 6A). This can be visualised in representative H&E images in Fig. 6D, indicating accelerated destruction of the alveolar tissue in the 2-hit cohort vs. 1-hit cohort. No significant differences in septal width were observed between any group at any timepoint (data not shown). Taken together, this is indicative of *P. aeruginosa* infection accelerating PPE-induced lung damage *in vivo.*

**Fig. 7:**
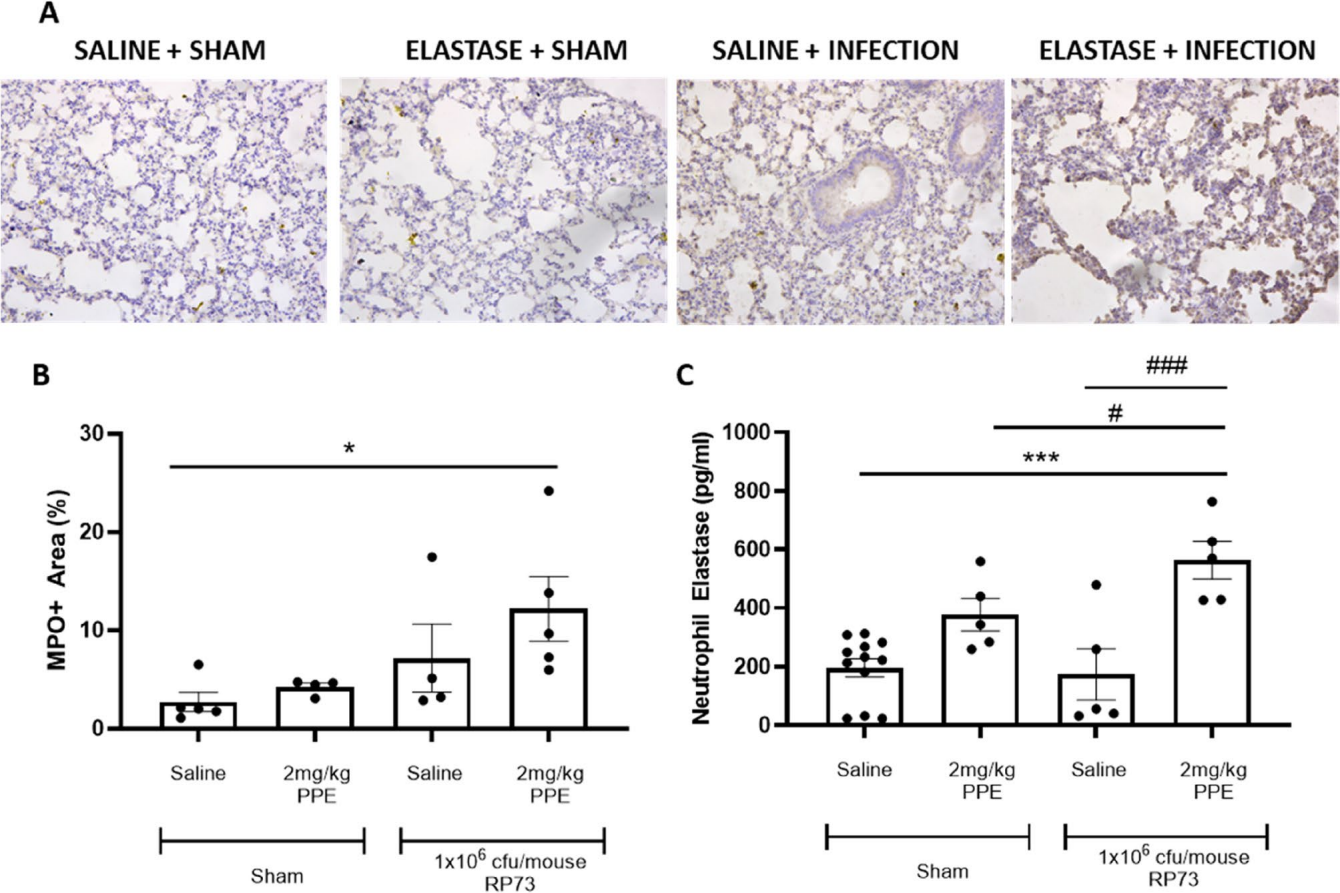
Pulmonary *P. aeruginosa* infection following intranasal PPE challenge demonstrates an increase in neutrophil specific protease release in the lung. C57Bl/6 mice were treated with 2 mg/kg PPE *i.n* on Days 1, 4 and 8; control animals were treated with vehicle only. 3-days post final aspiration animals were inoculated with 1 × 10^6^ cfu/mouse of the *P. aeruginosa* strain RP73 or saline control via *i.t* administration. Lungs were removed and fixed in 10% paraformaldehyde. Tissue sections were stained with an anti-MPO antibody for quantification of neutrophil-derived MPO release (**A**: representative images for MPO staining, **B**: Quantification of MPO staining). In other studies, a bronchoalveolar lavage was performed and analysed for NE by ELISA. *n* = 4–12. Data were analysed using a one-way ANOVA and Sidaks multiple comparisons post-hoc test. * = *P* < 0.05, *** = *P* < 0.001, compared to elastase challenge infected animals (column D). # = *P* < 0.05, ### = *P* < 0.001 compared between 1-hi and 2-hit cohorts (column B vs. D and C vs. D)

Following recruitment to the lungs, neutrophils are known to release a number of pro-inflammatory mediators including MPO and NE which are known to be associated with increased tissue damage. We therefore evaluated neutrophil derived MPO and NE 2-days post infection to investigate whether the accelerated rate of lung damage associated with the two-hit cohort was due to exacerbated neutrophil influx and activation. Figure 7A provides representative histological images of MPO staining, with significant elevations in MPO staining observed 2-days post infection in the 2-hit cohort compared to sham control (Fig. 7B, *P* < 0.05). Furthermore, NE levels present in BAL fluid were significantly elevated in the two-hit cohort compared with all other groups (Fig. 7C), suggesting that the significant elevations in neutrophil recruitment in the two-hit cohort were associated with increased protease release.

## Discussion

Animal models of pulmonary bacterial infection have been extensively used in both the efficacy evaluation of novel antimicrobials, and the investigation of host defence mechanisms to bacterial infection. However, despite the models replicating many pathophysiological characteristics of stable chronic infections, these models do not adequately replicate changes observed during bacterial PExs. In this study we have therefore sought to characterise a novel two-hit model of bacterial infection with the clinically relevant gram-negative bacteria *P. aeruginosa*. Many respiratory diseases associated with bacterial PExs are characterised by chronic neutrophil-dominated airways inflammation [1]. This excessive neutrophilic inflammation results in increased levels of MPO, NE and MMPs, resulting in extracellular matrix degradation, goblet cell metaplasia, mucus hypersecretion and increased airway damage [1]. As PPE has regularly been used to model COPD-like lung damage [31, 32, 38], we selected it as the agent to induce disease relevant lung damage prior to infection. Repeated aspiration of 2 mg/kg PPE induced significant airway damage compared to saline controls at day 11, as shown through significant pulmonary neutrophil recruitment, epithelial damage, alveolar wall breakdown, septal wall thickening, goblet cell metaplasia, and increases in the pro-inflammatory cytokines TNFα, IL-6, MCP-1 and KC (the mouse analogue of human IL-8). This observed pro-inflammatory phenotype was consistent with previously reported literature demonstrating lung inflammation and injury characteristic of the CF/COPD/NCFBE lung [29, 32, 39, 40].

Clinically, a key consequence of this chronic airway inflammation is reduced mucociliary clearance through epithelial cell damage and mucus hypersecretion leading to impaired bacterial clearance [1]. Current animal models of chronic infection where healthy animals are infected with *P. aeruginosa* usually require the immobilisation of bacteria in agar/alginate beads to prevent bacterial clearance. This is presumably due to effective mucociliary clearance and functional innate immune responses in healthy mice, as shown by our study here where in the absence of prior elastase challenge where *P. aeruginosa* was rapidly cleared over the 7-day time course of infection. Correspondingly, this was also associated with a similar resolution of neutrophil inflammation and pulmonary cytokine titres. However, in contrast, we demonstrated that bacterial clearance following intratracheal administration of a bacterial suspension was substantially impaired in animals subjected to repeated elastase challenge (two-hit cohort), with significant bacterial burden retained throughout the 7-day infection time-course. Importantly, the lungs of these animals demonstrated corresponding airway damage alongside goblet cell metaplasia induced by repeated challenge with 2 mg/kg PPE which is consistent with impaired mucociliary clearance observed in patients [1]. In addition to prolonged bacterial infection, pulmonary neutrophil recruitment remained significantly elevated in the 2-hit cohort throughout the duration of study compared to the 1-hit cohort. Murine models of pulmonary neutrophilic inflammation in response to bacteria derived stimuli such as LPS as also associated with increased circulating peripheral neutrophils alongside increased activation [41–43]. It is therefore possible that the initial elastase challenge may mobilise and activate circulating neutrophils thus priming them for more efficient pulmonary recruitment on the exposure to the second bacterial hit. This is supported by observations in clinical samples where patients with diseases including CF and NCFBE demonstrate an increase in both number and activation of peripheral neutrophils between both stable and exacerbating patients [44, 45]. However, additional research is required to evaluate this possibility further.

The ability of the animals to respond to this pulmonary infection was also affected as the mobilisation of the innate immune response appeared impaired. This was shown by reduced pulmonary inflammatory mediators including TNFα, IL-6, MCP-1 and KC compared to the one-hit cohort, and is consistent with the paradigmatic airways inflammation observed in respiratory diseases [11, 13, 46, 47]. Therefore, the combination of impaired mucociliary clearance and dysfunctional innate immune responses observed in our model enabled the onset of a tractable chronic pulmonary infection that is clinically relevant to CF, COPD and NCFBE patients.

It is widely accepted that chronic pulmonary infections in patients with underlying respiratory diseases are characterised by periods of stable infection, punctuated by periods of exacerbated infection. Whilst there remains no universally agreed definition of PExs, it is accepted that PExs triggered by either viral or bacterial infections are characterised by changes in baseline dyspnoea, cough, and/or sputum which is beyond normal daily variation [9, 14–17]. These exacerbations are associated with increases in airway inflammation, mucus secretion, pulmonary neutrophilia, and pro-inflammatory mediators including neutrophil elastase and TNFα [14, 16, 48, 49]. As our two-hit model of *P. aeruginosa* infection replicated an ‘acute-on-chronic’ phenotype with both neutrophilic inflammation and pathogens relevant to CF, COPD and NCFBE exacerbations, we sought to investigate whether our model also replicated key pathophysiological changes associated with acute bacterial PExs. Consistent with clinical observations of PExs, our model demonstrated that *P. aeruginosa* infection combined with PPE-induced lung injury resulted in a significant increase in airway neutrophilia that persisted for the duration of the study. This was also supported by a significant increase in MPO staining in lung tissue, alongside increased free neutrophil elastase and TNFα concentrations in BAL fluid 48 h post infection. Therefore, subsequent evaluation considered whether this exaggerated airway inflammation correlated with increased lung tissue damage. Singular challenge of 1 × 10^6^ cfu/mouse *P. aeruginosa* failed to demonstrate any prolonged lung inflammation or lung damage consistent with acute lung infection models at bacterial titres not associated with severe disease, whilst repeated PPE challenge alone induced progressive airway damage throughout the duration of the study consistent with previously reported literature [29, 32, 34]. Notably, the observed progressive lung damage measured by MLI [35] was significantly accelerated by secondary bacterial infection, with lung tissue damage peaking at 2-days post infection in the two-hit cohort vs. 7-days post infection following singular PPE challenge. Whilst our studies suggest that the accelerated lung injury was as a result of increased neutrophil recruitment and protease release, it remains one of many factors that may contribute to this accelerated damage, with other potential contributing factors including the release of endotoxins and other bacterial derived enzymes capable of inducing tissue damage. Therefore, further work is required to build on the data reported in this study.

These studies have focused on using the RP73 strain of *P.aeruginosa* isolated from a CF patient chronic infected with the pathogen. Whilst the use of this strain has provided significant evidence for infection mediated acceleration of neutrophilic lung injury, RP73 itself is considered to possess a low-inflammatory infectious phenotype due to its mucoid phenotype. Future work is therefore required to further investigate how other strains and species of bacteria clinically relevant to these respiratory diseases impact the exacerbation of PPE based lung injury.

## Conclusions

Whilst animal models of chronic respiratory infections have been utilised for the understanding of host immune responses to infection and the evaluation of novel antimicrobial therapies, currently no models exist that replicate key characteristics of PExs. Here for the first time, we demonstrate a two-hit model of pulmonary infection with *P. aeruginosa* that more accurately replicates the ‘acute-on-chronic’ phenotype associated with bacterial PExs. Significantly, this study highlights for the first time an in vivo model of infection that replicates key pathophysiologic changes exacerbated airway neutrophilic inflammation and accelerated lung damage which is consistent with acute PExs seen in CF, COPD and NCFBE patients.

## Data Availability

The datasets used and/or analysed during the current study are available from the corresponding author on reasonable request.

## Abbreviations

BAL: Bronchoalveolar lavage
CF: Cystic fibrosis
COPD: Chronic obstructive pulmonary disease
DAMP: Damage associated molecular pattern
*H. Influenzae*: Haemophilus influenzae
IL-6: Interleukin 6
IHC: Immunohistochemistry
KC: Keratinocyte chemoattractant
MCP-1: Monocyte chemoattractant protein-1
MLI: Mean linear intercept
MMP: Matrix metalloproteinase
MPO: Myeloperoxidase
MSW: Mean septum width
NCFBE: Non cystic fibrosis bronchiectasis
NE: Neutrophil elastase
*K.pneumoniae*: *Klebsiella pneumoniae*
*P.aeruginosa*: *Pseudomonas aeruginosa*
PAMP: Pattern associated molecular pattern
PBS: Phosphate buffered saline
PAS: Periodic acid-Schiff
PExs: Pulmonary exacerbations
PPE: Porcine pancreatic elastase
*S.aureus*: *Staphylococcus aureus*
*S.pneumonaie*: *Streptococcus pneumoniae*
TSA: Tryptic soy agar
TSB: Tryptic soy broth
TNFa: Tumour necrosis factor alpha

## References

[1] Fenker D, Cameron TM, Warunya P, Ralph JP, Eric JS, Carleen S et al. A Comparison between Two Pathophysiologically Different yet Microbiologically Similar Lung Diseases: Cystic Fibrosis and Chronic Obstructive Pulmonary Disease. Int J Respir Pulm Med [Internet]. 2018 Dec 31 [cited 2025 Feb 7];5(2). Available from: https://pubmed.ncbi.nlm.nih.gov/30627668/10.23937/2378-3516/1410098 PMC6322854 30627668

[2] Safiri S, Mahmoodpoor A, Kolahi AA, Nejadghaderi SA, Sullman MJM, Mansournia MA et al. Global burden of lower respiratory infections during the last three decades. Front public Heal [Internet]. 2023 Jan 9 [cited 2025 Feb 7];10. Available from: https://pubmed.ncbi.nlm.nih.gov/36699876/10.3389/fpubh.2022.1028525 PMC9869262 36699876

[3] Pahal P, Rajasurya V, Sharma S. Typical Bacterial Pneumonia. StatPearls [Internet]. 2023 Jul 31 [cited 2025 Feb 7]; Available from: https://www.ncbi.nlm.nih.gov/books/NBK534295/30485000

[4] Pragman AA, Berger JP, Williams BJ. Understanding persistent bacterial lung infections: Clinical implications informed by the biology of the microbiota and biofilms. Clin Pulm Med [Internet]. 2016 [cited 2025 Feb 7];23(2):57–66. Available from: https://journals.lww.com/clinpulm/fulltext/2016/03000/understanding_persistent_bacterial_lung.1.aspx 10.1097/CPM.0000000000000108 PMC4798234 27004018

[5] Blanchard AC, Waters VJ. Opportunistic Pathogens in Cystic Fibrosis: Epidemiology and Pathogenesis of Lung Infection. J Pediatric Infect Dis Soc. 2022;11:S3-12. 10.1093/jpids/piac052. 36069904 10.1093/jpids/piac052

[6] Leung JM, Tiew PY, Mac Aogáin M, Budden KF, Yong VFL, Thomas SS, et al. The role of acute and chronic respiratory colonization and infections in the pathogenesis of COPD. Respirology. 2017;22(4):634 https://pmc.ncbi.nlm.nih.gov/articles/PMC7169176/. 28342288 10.1111/resp.13032 PMC7169176

[7] Borekci S, Halis A, Aygun G, Musellim B. Bacterial colonization and associated factors in patients with bronchiectasis. Ann Thorac Med. 2016;11(1):55 https://pmc.ncbi.nlm.nih.gov/articles/PMC4748616/. 26933458 10.4103/1817-1737.172297 PMC4748616

[8] Patient Registry | Cystic Fibrosis Foundation [Internet]. [cited 2025 Feb 7]. Available from: https://www.cff.org/medical-professionals/patient-registry

[9] De Angelis A, Johnson ED, Sutharsan S, Aliberti S, Chalmers JD, Ringshausen FC, et al. Exacerbations of bronchiectasis. Eur Respir Rev. 2024;33(173):240085 https://pmc.ncbi.nlm.nih.gov/articles/PMC11267293/. 39048130 10.1183/16000617.0085-2024 PMC11267293

[10] Whitters D, Stockley R. Immunity and bacterial colonisation in bronchiectasis. Thorax. 2011;67(11):1006–13 https://europepmc.org/article/med/21933944. 21933944 10.1136/thoraxjnl-2011-200206

[11] Sethi S, Murphy TF. Infection in the pathogenesis and course of chronic obstructive pulmonary disease. N Engl J Med. 2008;359(22):2355–65 https://pubmed.ncbi.nlm.nih.gov/19038881/. 19038881 10.1056/NEJMra0800353

[12] Gellatly SL, Hancock REW. *Pseudomonas aeruginosa*: new insights into pathogenesis and host defenses. Pathog Dis. 2013;67(3):159–73. 10.1111/2049-632X.12033. 23620179 10.1111/2049-632X.12033

[13] Yonker LM, Cigana C, Hurley BP, Bragonzi A. Host-pathogen interplay in the respiratory environment of cystic fibrosis. J Cyst Fibros. 2015;14(4):431–9 https://pubmed.ncbi.nlm.nih.gov/25800687/. 25800687 10.1016/j.jcf.2015.02.008 PMC4485938

[14] Ritchie AI, Wedzicha JA, Definition. Definition. Causes, Pathogenesis, and Consequences of Chronic Obstructive Pulmonary Disease Exacerbations. Clin Chest Med. 2020;41(3):421–38 https://pubmed.ncbi.nlm.nih.gov/32800196/. 32800196 10.1016/j.ccm.2020.06.007 PMC7423341

[15] Stanford GE, Dave K, Simmonds NJ. Pulmonary Exacerbations in Adults With Cystic Fibrosis: A Grown-up Issue in a Changing Cystic Fibrosis Landscape. Chest. 2020;159(1):93 https://pmc.ncbi.nlm.nih.gov/articles/PMC7502225/. 32966813 10.1016/j.chest.2020.09.084 PMC7502225

[16] Wedzicha JA, Seemungal TA. COPD exacerbations: defining their cause and prevention. Lancet (London, England). 2007;370(9589):786–96 https://pubmed.ncbi.nlm.nih.gov/17765528/. 17765528 10.1016/S0140-6736(07)61382-8 PMC7134993

[17] Erfanimanesh S, Emaneini M, Modaresi MR, Feizabadi MM, Halimi S, Beigverdi R, et al. Distribution and Characteristics of Bacteria Isolated from Cystic Fibrosis Patients with Pulmonary Exacerbation. Can J Infect Dis Med Microbiol = J Can des Mal Infect la Microbiol Médicale. 2022;2022:5831139 https://pmc.ncbi.nlm.nih.gov/articles/PMC9805393/. 10.1155/2022/5831139 PMC9805393 36593975

[18] Domenech A, Puig C, Martí S, Santos S, Fernández A, Calatayud L, et al. Infectious etiology of acute exacerbations insevere COPD patients. J Infect. 2013;67(6):516–23 http://www.journalofinfection.com/article/S0163445313002557/fulltext. 24055804 10.1016/j.jinf.2013.09.003

[19] Bragonzi A. Murine models of acute and chronic lung infection with cystic fibrosis pathogens. Int J Med Microbiol. 2010;300(8):584–93. 20951086 10.1016/j.ijmm.2010.08.012

[20] Bielen K, Jongers B, Malhotra-Kumar S, Jorens PG, Goossens H, Kumar-Singh S. Animal models of hospital-acquired pneumonia: current practices and future perspectives. Ann Transl Med [Internet]. 2017 Mar 1 [cited 2025 Feb 7];5(6). Available from: https://pubmed.ncbi.nlm.nih.gov/28462212/10.21037/atm.2017.03.72 PMC5395468 28462212

[21] Evans SE, Tuvim MJ, Zhang J, Larson DT, García CD, Pro SM et al. Host lung gene expression patterns predict infectious etiology in a mouse model of pneumonia. Respir Res [Internet]. 2010 Jul 23 [cited 2025 Feb 7];11(1). Available from: https://pubmed.ncbi.nlm.nih.gov/20653947/10.1186/1465-9921-11-101 PMC2914038 20653947

[22] McConnell KW, McDunn JE, Clark AT, Dunne WM, Dixon DJ, Turnbull IR, et al. Streptococcus pneumoniae and Pseudomonas aeruginosa pneumonia induce distinct host responses. Crit Care Med. 2010;38(1):223 https://pmc.ncbi.nlm.nih.gov/articles/PMC2796712/. 19770740 10.1097/CCM.0b013e3181b4a76b PMC2796712

[23] Dudhani RV, Turnidge JD, Coulthard K, Milne RW, Rayner CR, Li J, et al. Elucidation of the pharmacokinetic/pharmacodynamic determinant of colistin activity against Pseudomonas aeruginosa in murine thigh and lung infection models. Antimicrob Agents Chemother. 2010;54(3):1117–24 https://journals.asm.org/journal/aac. 20028824 10.1128/AAC.01114-09 PMC2826009

[24] Facchini M, De Fino I, Riva C, Bragonzi A. Long term chronic Pseudomonas aeruginosa airway infection in mice. J Vis Exp [Internet]. 2014 Jan [cited 2015 Oct 16];(85). Available from: http://www.ncbi.nlm.nih.gov/pubmed/24686327 10.3791/51019 PMC4151694 24686327

[25] Amison RT, O’Shaughnessy BG, Arnold S, Cleary SJ, Nandi M, Pitchford SC, et al. Platelet Depletion Impairs Host Defense to Pulmonary Infection with *Pseudomonas aeruginosa* in Mice. Am J Respir Cell Mol Biol. 2018;58(3):331–40 http://www.ncbi.nlm.nih.gov/pubmed/28957635. 28957635 10.1165/rcmb.2017-0083OC

[26] Amison RT, Faure ME, O’Shaughnessy BG, Bruce KD, Hu Y, Coates A, et al. The small quinolone derived compound HT61 enhances the effect of tobramycin against Pseudomonas aeruginosa in vitro and in vivo. Pulm Pharmacol Ther. 2020;61:101884. 31887372 10.1016/j.pupt.2019.101884

[27] Gaggar A, Li Y, Weathington N, Winkler M, Kong M, Jackson P, et al. Matrix metalloprotease-9 dysregulation in lower airway secretions of cystic fibrosis patients. Am J Physiol - Lung Cell Mol Physiol. 2007;293(1):96–104. 10.1152/ajplung.00492.2006. 10.1152/ajplung.00492.2006 17384080

[28] Xu X, Abdalla T, Bratcher PE, Jackson PL, Sabbatini G, Wells JM, et al. Doxycycline improves clinical outcomes during cystic fibrosis exacerbations. Eur Respir J. 2017;49(4):1601102 https://publications.ersnet.org/content/erj/49/4/1601102. 28381428 10.1183/13993003.01102-2016

[29] Karandashova S, Kummarapurugu AB, Zheng S, Chalfant CE, Voynow JA. Neutrophil elastase increases airway ceramide levels via upregulation of serine palmitoyltransferase. Am J Physiol - Lung Cell Mol Physiol. 2018;314(1):L206-14. 10.1152/ajplung.00322.2017. 29025713 10.1152/ajplung.00322.2017 PMC5866429

[30] Serré J, Tanjeko AT, Mathyssen C, Vanherwegen AS, Heigl T, Janssen R, et al. Enhanced lung inflammatory response in whole-body compared to nose-only cigarette smoke-exposed mice. Respir Res. 2021;22(1):1–15 https://respiratory-research.biomedcentral.com/articles/. 33731130 10.1186/s12931-021-01680-5 PMC7968299

[31] Shimoyama T, Kaneda M, Yoshida S, Michihara S, Fujita N, Han LK, et al. Ninjin’yoeito ameliorated PPE-induced pulmonary emphysema and anxiety/depressive-like behavior in aged C57BL/6J mice. Front Pharmacol. 2022;13:970697 https://pmc.ncbi.nlm.nih.gov/articles/PMC9589273/. 36299904 10.3389/fphar.2022.970697 PMC9589273

[32] Oliveira MV, Abreu SC, Padilha GA, Rocha NN, Maia LA, Takiya CM, et al. Characterization of a Mouse Model of Emphysema Induced by Multiple Instillations of Low-Dose Elastase. Front Physiol. 2016;7:457 https://pmc.ncbi.nlm.nih.gov/articles/PMC5054025/. 27774071 10.3389/fphys.2016.00457 PMC5054025

[33] Bianconi I, Jeukens J, Freschi L, Alcalá-Franco B, Facchini M, Boyle B, et al. Comparative genomics and biological characterization of sequential Pseudomonas aeruginosa isolates from persistent airways infection. BMC Genomics. 2015;16(1):1105 http://www.ncbi.nlm.nih.gov/pubmed/26714629. 26714629 10.1186/s12864-015-2276-8 PMC4696338

[34] Arai N, Kondo M, Izumo T, Tamaoki J, Nagai A. Inhibition of neutrophil elastase-induced goblet cell metaplasia by tiotropiumin mice. Eur Respir J. 2010;35(5):1164–71 https://publications.ersnet.org/content/erj/35/5/1164. 19897560 10.1183/09031936.00040709

[35] Crowley G, Kwon S, Caraher EJ, Haider SH, Lam R, Batra P et al. Quantitative lung morphology: semi-automated measurement of mean linear intercept. BMC Pulm Med [Internet]. 2019 Nov 9 [cited 2025 Feb 7];19(1). Available from: https://pubmed.ncbi.nlm.nih.gov/31706309/10.1186/s12890-019-0915-6 PMC6842138 31706309

[36] Kukavica-Ibrulj I, Bragonzi A, Paroni M, Winstanley C, Sanschagrin F, O’Toole GA, et al. In vivo growth of Pseudomonas aeruginosa strains PAO1 and PA14 and the hypervirulent strain LESB58 in a rat model of chronic lung infection. J Bacteriol. 2008;190(8):2804–13. 10.1128/jb.01572-07. 18083816 10.1128/JB.01572-07 PMC2293253

[37] Jeukens J, Boyle B, Bianconi I, Kukavica-Ibrulj I, Tümmler B, Bragonzi A et al. Complete Genome Sequence of Persistent Cystic Fibrosis Isolate Pseudomonas aeruginosa Strain RP73. Genome Announc [Internet]. 2013 Jan [cited 2016 Feb 5];1(4). Available from: http://www.pubmedcentral.nih.gov/articlerender.fcgi?artid=3731849&tool=pmcentrez&rendertype=abstract 10.1128/genomeA.00568-13 PMC3731849 23908295

[38] Fysikopoulos A, Seimetz M, Hadzic S, Knoepp F, Wu CY, Malkmus K, et al. Amelioration of elastase-induced lung emphysema and reversal of pulmonary hypertension by pharmacological iNOS inhibition in mice. Br J Pharmacol. 2021;178(1):152–71. 32201936 10.1111/bph.15057

[39] Voynow JA, Fischer BM, Malarkey DE, Burch LH, Wong T, Longphre M et al. Neutrophil elastase induces mucus cell metaplasia in mouse lung. Am J Physiol Lung Cell Mol Physiol [Internet]. 2004 Dec [cited 2025 Feb 7];287(6). Available from: https://pubmed.ncbi.nlm.nih.gov/15273079/10.1152/ajplung.00140.2004 15273079

[40] Taguchi L, Pinheiro NM, Olivo CR, Choqueta-Toledo A, Grecco SS, Lopes FDTQS, et al. A flavanone from *Baccharis retusa* (Asteraceae) prevents elastase-induced emphysema in mice by regulating NF-κB, oxidative stress and metalloproteinases. Respir Res. 2015;16(1): 79. 10.1186/s12931-015-0233-3. 26122092 10.1186/s12931-015-0233-3 PMC4489216

[41] Kornerup KN, Salmon GP, Pitchford SC, Liu WL, Page CP. Circulating platelet-neutrophil complexes are important for subsequent neutrophil activation and migration. J Appl Physiol. 2010;109(3):758–67 http://www.ncbi.nlm.nih.gov/pubmed/20558756. 20558756 10.1152/japplphysiol.01086.2009

[42] Cleary SJ, Hobbs C, Amison RT, Arnold S, O’Shaughnessy BG, Lefrancais E, et al. LPS-induced Lung Platelet Recruitment Occurs Independently from Neutrophils, PSGL-1, and P-Selectin. Am J Respir Cell Mol Biol. 2019;61(2):232–43 http://www.ncbi.nlm.nih.gov/pubmed/30768917. 30768917 10.1165/rcmb.2018-0182OC PMC6670039

[43] Pan D, Amison RT, Riffo-Vasquez Y, Spina D, Cleary SJ, Wakelam MJ, et al. P-Rex and Vav Rac-GEFs in platelets control leukocyte recruitment to sites of inflammation. Blood. 2014;125(7):1146–58 http://www.pubmedcentral.nih.gov/articlerender.fcgi?artid=4326774%26;tool=pmcentrez%26;rendertype=abstract. 25538043 10.1182/blood-2014-07-591040 PMC4326774

[44] Daniel P, Ashton D, Sheppard C, Eletu S, Sandu P, Litt D, et al. S106 Peripheral blood neutrophils are primed and activated in bronchiectasis and are attenuated by the pro-resolving mediator lipoxin a4. Thorax. 2016;71(3):A62-3 https://thorax.bmj.com/content/71/Suppl_3/A62.2.

[45] Martin C, Dhôte T, Ladjemi MZ, Andrieu M, Many S, Karunanithy V, et al. Specific circulating neutrophils subsets are present in clinically stable adults with cystic fibrosis and are further modulated by pulmonary exacerbations. Front Immunol. 2022;13:1012310. 36248793 10.3389/fimmu.2022.1012310 PMC9560797

[46] Sethi S, Maloney J, Grove L, Wrona C, Berenson CS. Airway Inflammation and Bronchial Bacterial Colonization in Chronic Obstructive Pulmonary Disease. Am J Respir Crit Care Med. 2006;173(9):991 https://pmc.ncbi.nlm.nih.gov/articles/PMC2662918/. 16474030 10.1164/rccm.200509-1525OC PMC2662918

[47] Dente FL, Bilotta M, Bartoli ML, Bacci E, Cianchetti S, Latorre M et al. Neutrophilic Bronchial Inflammation Correlates with Clinical and Functional Findings in Patients with Noncystic Fibrosis Bronchiectasis. Mediators Inflamm [Internet]. 2015 [cited 2025 Feb 7];2015. Available from: https://pubmed.ncbi.nlm.nih.gov/26819500/10.1155/2015/642503 PMC4706949 26819500

[48] Goss CH. Acute pulmonary exacerbation in cystic fibrosis. Semin Respir Crit Care Med. 2019;40(6):792 https://pmc.ncbi.nlm.nih.gov/articles/PMC7528649/. 31659730 10.1055/s-0039-1697975 PMC7528649

[49] Dong K, Huh SM, Lam GY, Jang J, Franciosi AN, Wilcox PG, et al. Pulmonary exacerbation inflammatory phenotypes in adults with cystic fibrosis. J Cyst Fibros. 2023;22(2):306–12. 36572614 10.1016/j.jcf.2022.12.013

